# Electrically Controlled Liquid Crystal Microlens Array Based on Single-Crystal Graphene Coupling Alignment for Plenoptic Imaging

**DOI:** 10.3390/mi11121039

**Published:** 2020-11-26

**Authors:** Mingce Chen, Qi Shao, Wenda He, Dong Wei, Chai Hu, Jiashuo Shi, Kewei Liu, Haiwei Wang, Changsheng Xie, Xinyu Zhang

**Affiliations:** 1 National Key Laboratory of Science & Technology on Multispectral Information Processing, Huazhong University of Science & Technology, Wuhan 430074, China; D201780651@hust.edu.cn (M.C.); M201872585@hust.edu.cn (Q.S.); M201872714@hust.edu.cn (W.H.); D201677599@hust.edu.cn (D.W.); D201880681@hust.edu.cn (C.H.); D201980727@hust.edu.cn (J.S.); D202080878@hust.edu.cn (K.L.); 2 School of Artificial Intelligence and Automation, Huazhong University of Science & Technology, Wuhan 430074, China; 3 Innovation Insititute, Huazhong University of Science and Technology, Wuhan 430074, China; 4 Wuhan National Laboratory for Optoelectronics, Huazhong University of Science & Technology, Wuhan 430074, China; hiway@hust.edu.cn (H.W.); Cs_xie@hust.edu.cn (C.X.)

**Keywords:** liquid crystal (LC) device, single-crystal graphene (SCG) alignment, plenoptic imaging

## Abstract

As a unique electric-optics material, liquid crystals (LCs) have been used in various light-control applications. In LC-based light-control devices, the structural alignment of LC molecules is of great significance. Generally, additional alignment layers are required for LC lens and microlens, such as rubbed polyimide (PI) layers or photoalignment layers. In this paper, an electrically controlled liquid crystal microlens array (EC-LCMLA) based on single-crystal graphene (SCG) coupling alignment is proposed. A monolayer SCG with high conductivity and initial anchoring of LC molecules was used as a functional electrode, thus no additional alignment layer is needed, which effectively simplifies the basic structure and process flow of conventional LCMLA. Experiments indicated that a uniform LC alignment can be acquired in the EC-LCMLA cell by the SCG coupling alignment effect. The common optical properties including focal lengths and point spread function (PSF) were measured experimentally. Experiments demonstrated that the proposed EC-LCMLA has good focusing performance in the visible to near-infrared range. Moreover, the plenoptic imaging in Galilean mode was achieved by integrating the proposed EC-LCMLA with photodetectors. Digital refocusing was performed to obtain a rendering image of the target.

## 1. Introduction

**Figure 1:**
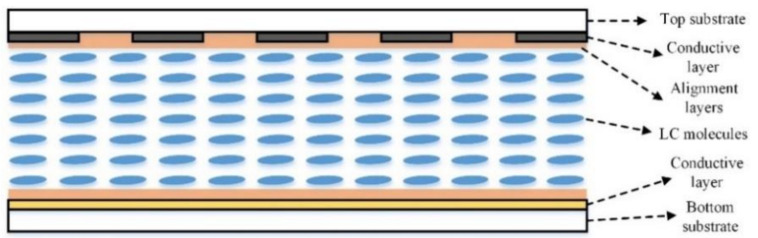
The general structure of liquid crystal microlens array (LCMLA), which includes top and bottom substrates with planar or patterned conductive layers, alignments layers, and a layer of LC molecules.

Owing to the excellent electric-optics characteristic of liquid crystal (LC) materials, the focal length of the LC-based lens and microlens can be electrically adjusted within a certain range. Moreover, they also have advantages of miniaturization, low power consumption and easy integration with other devices [1,2,3,4,5,6]. Therefore, LC-based lenses and microlenses have been widely used in 3D display [7,8,9,10,11,12], light-field cameras [13,14,15,16,17], 2D/3D tunable imaging [14,17,18,19] and beam steering [20,21,22,23]. As shown in Figure 1, the structure of liquid crystal microlens array (LCMLA) generally includes top and bottom substrates with planar or patterned conductive layers, alignments layers, and a layer of LC molecules sandwiched between them. When an appropriate driving voltage signal is applied on the conductive layers, gradient refractive index profiles of the extraordinary ray are formed with the redistribution of LC molecules. Therefore, a lens-like effect can be achieved [17].

Materials with high transmittance are used as top and bottom substrates to ensure sufficient light energy utilization. Quartz glass and zinc selenide (ZnSe) are the most commonly used in the visible and infrared bands [24,25,26,27,28], respectively. Aluminum (Al) and indium-tin-oxide (ITO) are commonly used as conductive layers (i.e., the electrodes of LCMLA) for applying an external electric field, which results in refractive index modulation (bi-refringence). Alignment layers such as rubbed polyimide(PI) layers and photoalignment layers [29,30] are used to realize an LC uniform alignment for ensuring the device’s performance. Inevitably, these alignment layers will reduce the transmittance of available light. Due to the advantages of high stability, excellent reliability and suitability for large-area processing, rubbed PI layers are the most commonly used. Both the micro-grooves and polymer chains formed in rubbed PI layers facilitate the uniform alignment of LC molecules. Typically, the micro-grooves have a depth in sub-micrometer scale and a width in micrometer scale for initially aligning the LC molecules contacted directly with them [31,32]. So far, the average depth and width of the micro-grooves formed by rubbing PI films for presenting an ideal light control performance have been decreased in several tens of the nanometer and sub-micron scale, respectively. A typical value of the average interval between adjacent micro-grooves is ~0.7 μm [33], such an orderly micro-groove arrangement usually causes diffraction crosstalk in the IR regime [34,35,36]. As an ultrathin flexible nanomaterial with high transparency in the visible and infrared range and excellent electrical conductivity, graphene has demonstrated several merits over a wide range of optoelectronic applications, such as broadband image sensors throughout the visible and infrared wavelengths, even extending to terahertz photodetection [36,37,38]. In addition, graphene can serve as an effective 2D crystalline template, which enables directional assembly of organic and inorganic nanomaterials via van der Waals interactions at the interface, resulting in improved functionalities of ordered nanomaterials. Especially, a strong and ordered organic LC molecule alignment can be obtained on conductive graphene, which provides clear advantages for LC display and other LC-based electric-optics devices [39,40,41]. In 2018, Shen et al. demonstrated that the single-crystal graphene (SCG) surface does not have an intrinsic preferential direction for epitaxial alignment of LC molecules (among the threefold symmetric easy axes), while the first contact geometry determines the alignment direction [42]. Despite the anchoring energy on SCG is strong, the LC alignment direction is readily erasable and rewritable. The LC alignment stability on SCG was proved to be excellent by one-year storge. After one-year of storge in a Petri dish on air, the LC alignment direction on SCG was identical, only the location of the opposite tilting domain boundary and the color had been changed, these undesired changes were usually expected due to the instability of cell thickness. Thus, the LC alignment stability on SCG is expected to be excellent when the thickness is sustained by spacers. In addition, the capillary injection method was proved to induce a uniform LC alignment on SCG.

In this paper, an alignment-layer-free electrically controlled liquid crystal microlens array(EC-LCMLA) without any mechanical rubbing was proposed and experimentally demonstrated. Compared with the conventional LCMLA using rubbed PI layers, the basic structure and process flow were effectively simplified. Moreover, the electrostatic accumulation, particle contamination and IR diffraction crosstalk introduced by mechanical rubbing could be effectively avoided. Experiments indicated that a uniform LC alignment can be acquired in the EC-LCMLA cell by SCG coupling alignment. Its common optical properties including focal lengths and point spread functions (PSFs) were experimentally analyzed. Experiments demonstrated that the proposed EC-LCMLA has good focusing performance in the visible to near-infrared range. Moreover, the plenoptic imaging in Galilean mode was achieved by integrating the proposed EC-LCMLA with a complementary metal-oxide semiconductor (CMOS) photodetector.

## 2. Materials and Methods

### 2.1. Structure of Electrically Controlled Liquid Crystal Microlens Array (EC-LCMLA)

**Figure 2:**
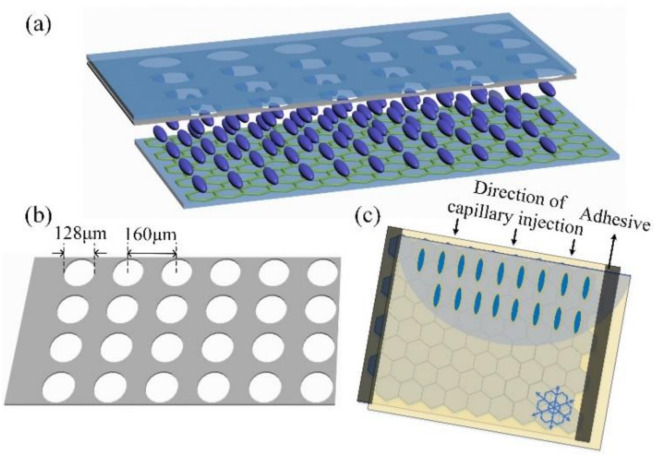
(**a**) The schematic of the proposed electrically controlled liquid crystal microlens array (EC-LCMLA). (**b**) The structure parameters of the patterned Al electrode. (**c**) Capillary injection process of LC molecules, noting that the patterned Al electrode was not shown.

The schematic of EC-LCMLA is depicted in Figure 2a. The key functional structures of the proposed EC-LCMLA are two ~500 μm silica substrates with different conductive films pre-coated over their inner surface. The inner surface of the top substrate was deposited with 100-nm-thick Al film by magnetron sputtering. After conventional ultraviolet photolithography and wet-etching process, the patterned Al electrode with an arrayed micro-holes was formed. As shown in Figure 2b, the diameter of micro-holes is 128 μm and the center-to-center distance is 160 μm. The bottom substrate was pre-transferred with monolayer SCG on its inner surface, which serves as both transparent eletrode and alignment layer (purchased from Beijing Graphene Insititute, Beijing, China). The transfer process was as follows: Firstly, a single crystal α-Al_2_O_3_ (0001) substrate (sapphire) was used as the epitaxial substrate, and a 500-nm-thick Cu film was deposited onto the sapphire substrate by magnetron sputtering. The Cu thin film was then annealed at 1000 °C at atmospheric pressure with hydrogen and argon to recrystallize and transform the sample into single crystal Cu(111). Highly oriented graphene domains were then grown on the Cu(111) substrate via atmospheric pressure CVD (APCVD). Subsequently, the oriented graphene domains were seamlessly stitched to form a SCG monolayer wafer. Finally, the monolayer SCG was transferred onto the silica substrate by clean transfer method. One of the three easy axes of the SCG is parallel to the short side of the SCG-silica substrate. Glass microsphere spacers of 20 μm diameter, mixed with the adhesive, were deposited to separate the two substrates. Finally, a layer of long rod-shaped nematic LC materials (E44 of Merck) was filled in the formed micro-cavity by capillary injection method. The electro-optical parameters are: n_e_ = 1.7904 and n_o_ = 1.5277 (∆n = 0.2627 at 589.3 nm, +20 °C) and ε_⊥_ = 5.2, ε_∥_ = 22.0, where ε_⊥_ and ε_∥_ are the dielectric constants of the LC molecules perpendicular or parallel to the director, respectively. Figure 2c shows the capillary injection process of LC molecules. Firstly, LC was dropped on an edge (one of the long sides) of the cell, and then LC molecules fully filled the cell owing to capillary effect, note that the two short sides of the LC cell were adhesively sealed before injection. During this process, the flow caused by capillary effect and the three easy axis directions of SCG codetermined the first contact geometry between LC molecules and SCG. Since the injection direction was parallel to the short side of the SCG-silica substrate (i.e., the selected easy axis direction), such a first contact geometry could induce an uniform LC alignment along this direction.

**Figure 3:**
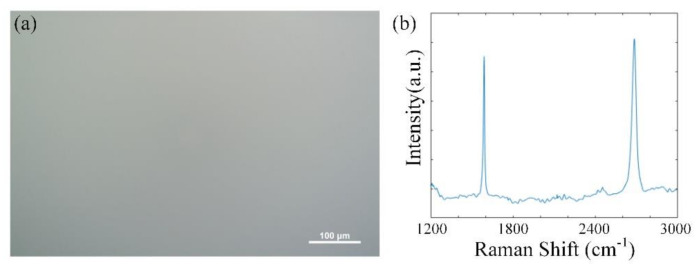
(**a**) The optical microscopy image of monolayer single-crystal graphene (SCG) on silica substrate. (**b**) Raman spectrum of the SCG-silica substrate.

The quality of monolayer SCG on silica substrate was checked by optical microscopy image and Raman spectrum, as shown in Figure 3. The optical microscopy image indicated that a fully covered SCG monolayer was transferred onto the silica substrate without any indication of multilayer regions. There is no detectable defect band according to the Raman spectrum.

### 2.2. Plenoptic Imaging System Based on EC-LCMLA

**Figure 4:**
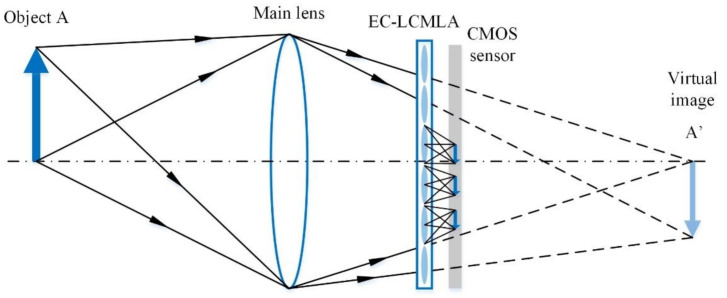
The schematic of the plenoptic imaging system based on EC-LCMLA working in Galilean mode.

The schematic of the proposed plenoptic imaging system is shown in Figure 4. The main lens and EC-LCMLA constitute two imaging subsystems. Firstly, incident light from object A is compressed by main lens, and the main lens tends to form images A’, which is behind the CMOS sensor without considering the EC-LCMLA, so the images A’ can be treated as virtual image, in which case it is known as the Galilean mode. Secondly, virtual image A’ is projected by LC microlenses as objective and, finally, an array of elemental images is formed on the CMOS sensor. Elemental images from adjacent LC microlenses are partially overlapping.

## 3. Experiments and Results

### 3.1. LC Molecules Alignment of EC-LCMLA

**Figure 5:**
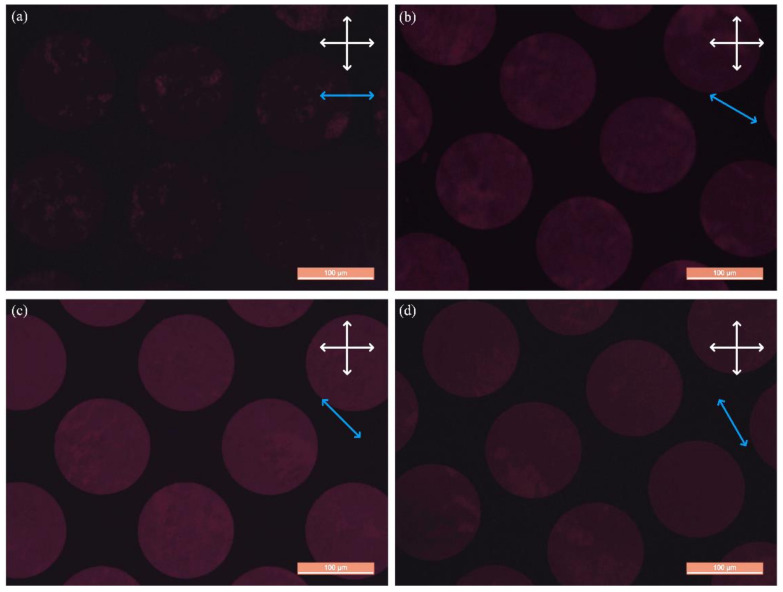
Optical characterization of the LC molecules alignment of EC-LCMLA. (**a**) In the initial state, the short side of EC-LCMLA (i.e., the selected easy axis direction) was parallel to the transmission axis of polarizer. (**b**–**d**) The captured polarized optical microscope (POM) images when the EC-LCMLA rotated by 30°, 45° and 60°, respectively. The white cross arrows represent the transmission axis directions of the analyzer and the polarizer, and the blue arrow represents the short side of the SCG-silica substrate (i.e., the selected easy axis direction).

A polarized optical microscope (DM4000 of Leica Microsystems, Wetzlar, Germany) was used to examine the uniform LC molecule alignment in the EC-LCMLA cell. During the experiments, the analyzer and the polarizer remained perpendicular to each other, the EC-LCMLA cell was placed between them. Polarized optical microscope (POM) images of EC-LCMLA cell were captured while rotating the EC-LCMLA cell. In the initial state, the short side of EC-LCMLA (i.e., the selected easy axis direction) was parallel to the transmission axis of the polarizer. The captured POM images are shown in Figure 5. White cross arrows represent the transmission axis directions of the analyzer and the polarizer, and the blue arrow represents the short side of the SCG-silica substrate (i.e., the selected easy axis direction).

As shown in Figure 5, when the selected easy axis direction was parallel to the transmission axis of the polarizer (i.e., the initial state), a uniform dark state appeared within the micro-holes region. When the EC-LCMLA was rotated by 30°, 45° and 60°, the optical axis of LC molecules and the polarizer transmission axis form acute angles of 30°, 45° and 60°, respectively. Therefore, the micro-holes region became uniformly bright. Among the three angles, the field of view corresponding to 45° was the brightest, and the brightness corresponding to 30° and 60° were roughly equivalent. Experiments indicated that a uniform LC alignment can be acquired in EC-LCMLA cell by SCG coupling alignment, and the LC alignment direction was parallel to the short side of the SCG substrate (i.e., the selected easy axis direction).

### 3.2. Optical Properties of EC-LCMLA

**Figure 6:**
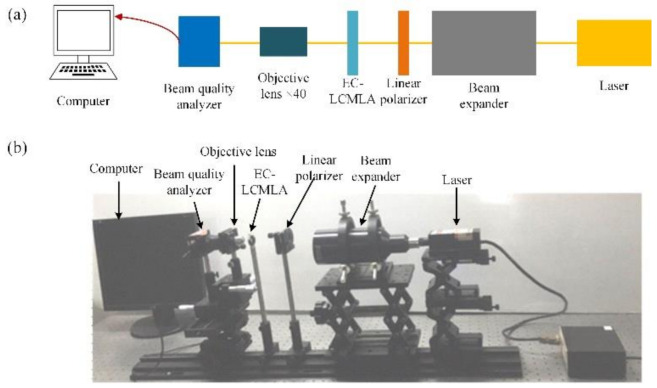
Optical measurement system for characterizing the proposed EC-LCMLA. (**a**) Measurement schematic diagram. (**b**) Actual testing platform.

To characterize the typical optical properties of the EC-LCMLA, a measurement system was constructed, as shown in Figure 6. When a voltage signal is applied, the relatively accurate value of focal length can be obtained by measuring the sharpest light intensity distribution [17]. A green laser with a wavelength of 501–561 nm (Changchun New Industries Optoelectronics Tech. Co., Ltd., Changchun, China) was firstly expanded by a beam expander (Newport, HB-20XAR) and then polarized by a linear polarizer (USP-50C0.4-38 of OptoSigma, Tokyo, Japan). Continuously, the polarized beam passed through the EC-LCMLA. The transmission axis of the linear polarizer was parallel to the alignment direction of LC molecules (i.e., the selected easy axis direction). Then, the light-fields were remarkably amplified by a microscope objective lens of ×40 and 0.65 numerical aperture and finally captured by a laser beam profiler (WinCamD of DataRay, Inc., Redding, CA, USA). To finely locate the focal planes shaped, we adjusted precisely the distance between the EC-LCMLA and the microscope objective for obtaining the sharpest light intensity distribution of the converged light-fields. The relatively accurate value of focal length is equal to the sum of the thickness of the silica substrate and the distance between the exiting end of EC-LCMLA and the incident surface of the microscope objective. Using the same method, the focal length and PSF corresponding to 980 nm (Changchun New Industries Optoelectronics Tech. Co., Ltd., Changchun, China) was measured. Note that the beam expander and polarizer were removed when performing tests of 980 nm.

**Figure 7:**
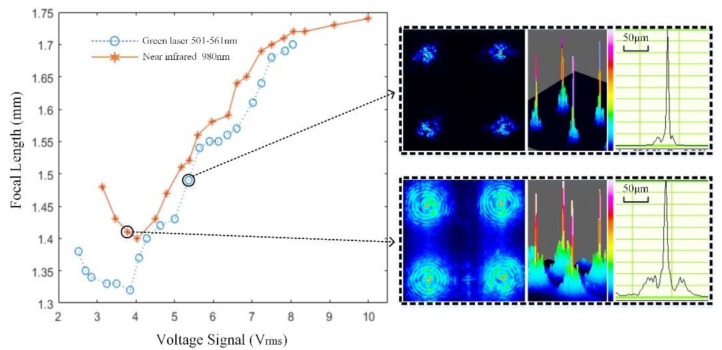
Relationship between the focal length of the proposed EC-LCMLA and root mean square (RMS) value of applied voltage signals. Figures in the dotted box indicate the 2D and 3D point spread functions (PSFs).

Figure 7 demonstrates the relationship between the focal length of the EC-LCMLA and the root mean square (RMS) value of applied voltage signals. The applied voltage signal was an AC square wave with a frequency of 1 kHz. Figures in the dotted box indicate the point spread functions (PSFs) of EC-LCMLA. For the green laser (501–561 nm), the actual full-width at half-maxima (FWHM) of the focal spot was between ~3 and ~4 μm. For the infrared laser (980 nm), the FWHM was between ~7 and ~8 μm. According to the experiments, the current EC-LCMLA could work well both in the visible (501–561 nm) and infrared range (980 nm), and the focusing performance and electrical tunability were excellent. Moreover, the relationship between focal length and voltage signals was similar to previous research [3,16,17].

### 3.3. Plenoptic Imaging Based on EC-LCMLA

**Figure 8:**
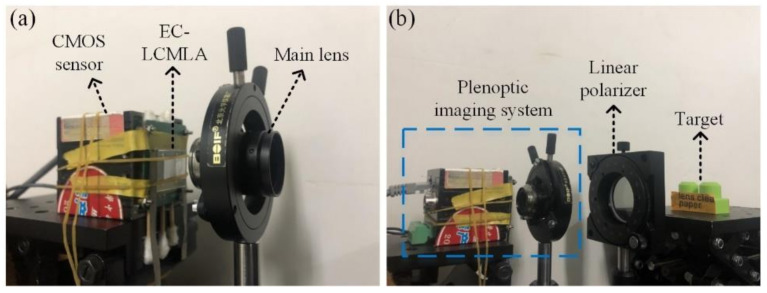
Photographs of: (**a**) The prototype of plenoptic imaging system, and (**b**) the experimental diagram for plenoptic imaging.

The prototype of the plenoptic imaging system and the experiment diagram are illustrated in Figure 8. As shown in Figure 8a, the plenoptic imaging system consists of a CMOS sensor, a fabricated EC-LCMLA, and the main lens with a focal length of 35 mm (M3520-MPW2 of Computar, Tokyo, Japan). The CMOS sensor (MVC14KSAC-GE6 of Microview, Beijing, China) was 4384 × 3288 pixels with a pixel pitch of 1.4 μm. The F-number of the main lens was set as 5.6 to match that of EC-LCMLA, which can achieve relatively full use of CMOS resolution while avoiding sub-images crosstalk.

Figure 8b demonstrates the experimental diagram for plenoptic imaging. An imaging target was placed 180 mm away from the plenoptic imaging system. The linear polarizer (USP-50C0.4-38 of OptoSigma, Tokyo, Japan) between them was used to satisfy the polarization sensitivity of the EC-LCMLA, thereby eliminating stray-light crosstalk. The transmission axis of the linear polarizer was parallel to the short side of the EC-LCMLA substrate (i.e., the selected easy axis direction). During experiments, the EC-LCMLA was driven by a square wave voltage signal with a frequency of 1 kHz. The voltage signals are generated by an AC generator.

**Figure 9:**
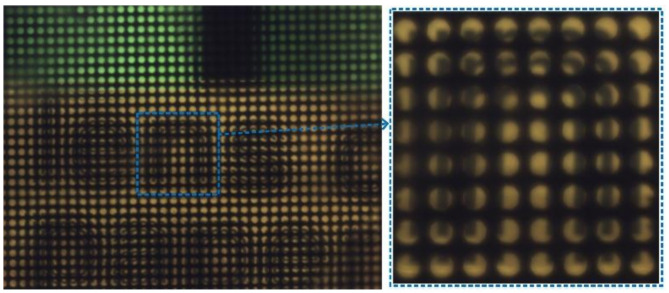
The raw light-field image and partially enlarged image captured by the plenoptic imaging system based on EC-LCMLA, the applied voltage signal is ~5.50 V_rms_. The image indicates obvious plenoptic imaging in Galilean mode.

Figure 9 shows the raw light-field image captured by the proposed plenoptic imaging system, the applied voltage signal was ~5.50 V_rms_. The dotted frame on the right side of Figure 9 is a partially enlarged image, which indicates obvious plenoptic imaging in Galilean mode [17,36].

**Figure 10:**
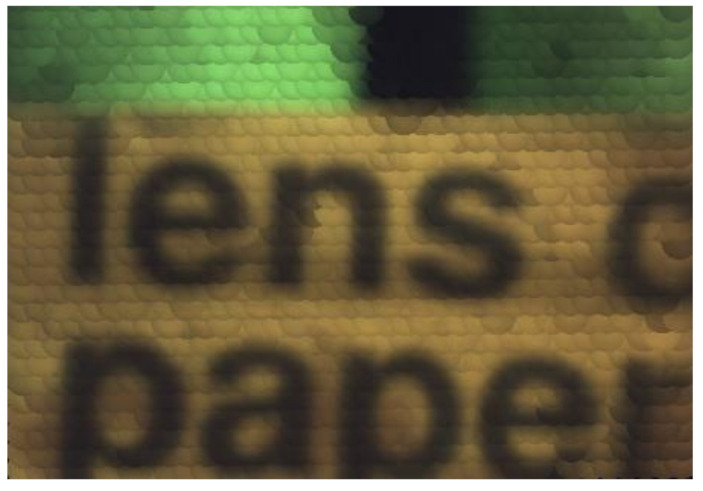
The rendering image corresponding to the captured raw light-field image.

Different from the traditional mechanical focusing method, digital refocusing relies entirely on digital calculation. By projecting the acquired 4D light-field to different image planes for integral superposition, the in-focus images of different image planes can be obtained. In the experiments, digital refocusing was performed based on the raw light-field image in Figure 9 to obtain a rendering image of the target [17]. Figure 10 shows the rendering image of the target.

## 4. Conclusions

In summary, we proposed a novel EC-LCMLA based on the coupling alignment of SCG. A monolayer of SCG with high conductivity and initial anchoring of LC molecules was used as a functional electrode, thus no additional alignment layer was needed, which can effectively simplify the basic structure and the process flow of conventional LCMLA. POM experiments indicated that a uniform LC alignment can be acquired in EC-LCMLA cell by SCG coupling alignment. Its common optical properties including focal lengths and PSFs were experimentally analyzed. Experiments demonstrated that the proposed EC-LCMLA has excellent focusing performance and electrical tunability in the visible and near-infrared range. Moreover, the plenoptic imaging in Galilean mode was achieved by integrating the proposed EC-LCMLA with a CMOS sensor.
